# Invasive *Lactuca serriola* seeds contain endophytic bacteria that contribute to drought tolerance

**DOI:** 10.1038/s41598-021-92706-x

**Published:** 2021-06-25

**Authors:** Seorin Jeong, Tae-Min Kim, Byungwook Choi, Yousuk Kim, Eunsuk Kim

**Affiliations:** School of Earth Sciences and Environmental Engineering, Gwangju Institute of Science and Technology, Gwangju, South Korea

**Keywords:** Invasive species, Bacterial host response, Plant symbiosis

## Abstract

The mutualistic relationship between alien plant species and microorganisms is proposed to facilitate or hinder invasive success, depending on whether plants can form novel associations with microorganisms in the introduced habitats. However, this hypothesis has not considered seed endophytes that would move together with plant propagules. Little information is available on the seed endophytic bacteria of invasive species and their effects on plant performance. We isolated the seed endophytic bacteria of a xerophytic invasive plant, *Lactuca serriola*, and examined their plant growth-promoting traits. In addition, we assessed whether these seed endophytes contributed to plant drought tolerance. Forty-two bacterial species were isolated from seeds, and all of them exhibited at least one plant growth-promoting trait. *Kosakonia cowanii* occurred in all four tested plant populations and produced a high concentration of exopolysaccharides in media with a highly negative water potential. Notably, applying *K. cowanii* GG1 to *Arabidopsis thaliana* stimulated plant growth under drought conditions. It also reduced soil water loss under drought conditions, suggesting bacterial production of exopolysaccharides might contribute to the maintenance of soil water content. These results imply that invasive plants can disperse along with beneficial bacterial symbionts, which potentially improve plant fitness and help to establish alien plant species.

## Introduction

Endophytic bacteria are non-pathogenic microbes that occur within plant tissues, including the stems, seeds, leaves, and fruits^1,2^. Endophytic bacteria have received considerable attention because they stimulate plant growth by producing plant growth-promoting (PGP) molecules or increasing plant resistance to biotic and abiotic environmental stresses^3–6^. Endophytes occurring inside seeds are of particular interest because they can be vertically transmitted from parents to offspring, effectively behaving as additional genes in their host^7^. Although seed endophytic bacteria from crop plant species and their application in agricultural activities have been widely investigated^8,9^, relatively little information is available on the ecological significance of these bacteria^7,10^.

Diverse abiotic and biotic factors determine the invasiveness of non-native plant species^11,12^. An important attribute of invasive success is the development of mutualistic relationships between invasive plants and microorganisms^13,14^. As invasive plants tend to disperse across a broad geographic range and colonize novel habitats, previous studies have stressed that the contribution of mutualism to invasive success would be highly dependent on the presence of novel mutualistic bacteria in the introduced habitats^13,15,16^. However, it should be noted that seed endophytic bacteria can disperse along with the host plants^17^. It is possible that seed endophytic bacteria improve the fitness of invasive plants, similar to the bacteria occurring in the rhizosphere or leaves ^18–20^. Invasive plants might benefit from seed endophytic bacteria instead of bacteria in the introduced habitats for their successful establishment.

Here, we isolated and characterized the seed endophytic bacteria of an invasive plant species, *Lactuca serriola* (Asteraceae). *L. serriola* is an annual or biennial herbaceous plant species that originated in Europe. Since it was first reported as an alien species in 1978, its range has expanded rapidly across South Korea^21,22^. The high drought tolerance of *L. serriola* has been suggested as a critical trait facilitating its successful invasion of open habitats, including roadsides, vacant lots, and ruins^21,23,24^.

We examined if seed endophytic bacteria produced PGP molecules, which are believed to promote plant growth^25^. In addition, we tested if these bacteria contributed to the drought tolerance of host plants. It has previously been demonstrated that bacterial production of exopolysaccharides (EPS) plays a critical role in increasing the drought tolerance of plants in addition to their effects on microbial physiology^26^. In particular, EPS-producing rhizobacteria efficiently colonize plant roots and aggregate rhizosphere soil by increasing the inter-particle cohesion forces among the soil particles, thereby increasing soil porosity and retention time of soil moisture^27–29^. Consequently, they can increase the adhering soil-to-root tissue ratio and alleviate water stress. We evaluated the EPS produced by the isolated seed endophytes and assessed if seed endophyte infection increased the drought tolerance of a model plant species, *Arabidopsis thaliana*.

## Results

### Identification of seed endophytic bacteria and their PGP traits

**Figure 1:**
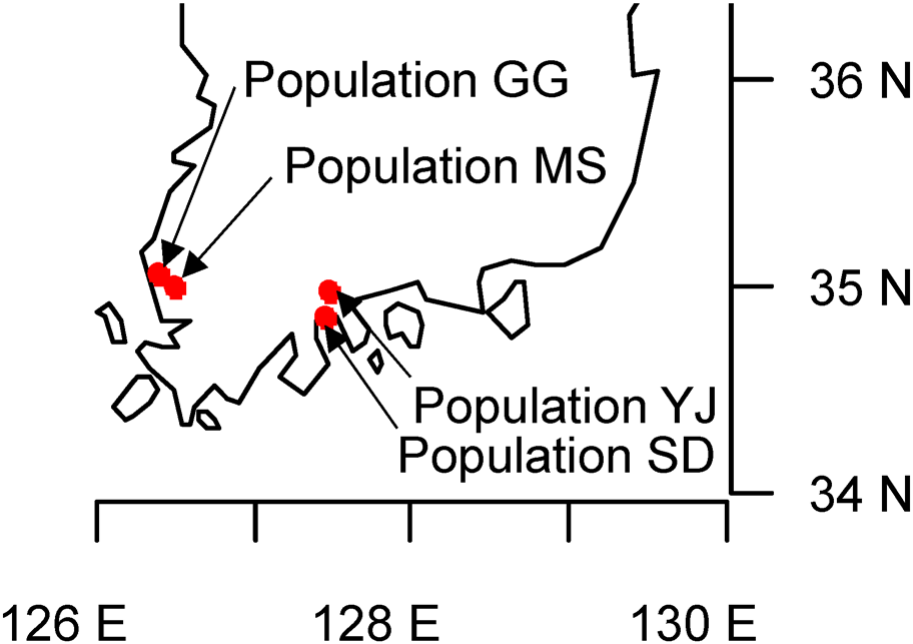
Sampling sites of *Lactuca serriola*. Seeds were collected from two distant geographical regions (Muan-gun and Suncheon-si) with two sampling sites each. The map was generated using maps package in R (Version 4.0.1, https://www.cran.r-project.org/web/packages/maps/maps.pdf).

We collected wild *L. serriola* seeds from four natural populations in the vicinity of the Gwangju Institute of Sciences and Technology, Gwangju, South Korea (Fig. 1, Supplementary Table S1). Seeds from 4–12 plants from each population were composited, and 80 seeds from each composite sample were used to isolate endophytic bacteria.

**Figure 2:**
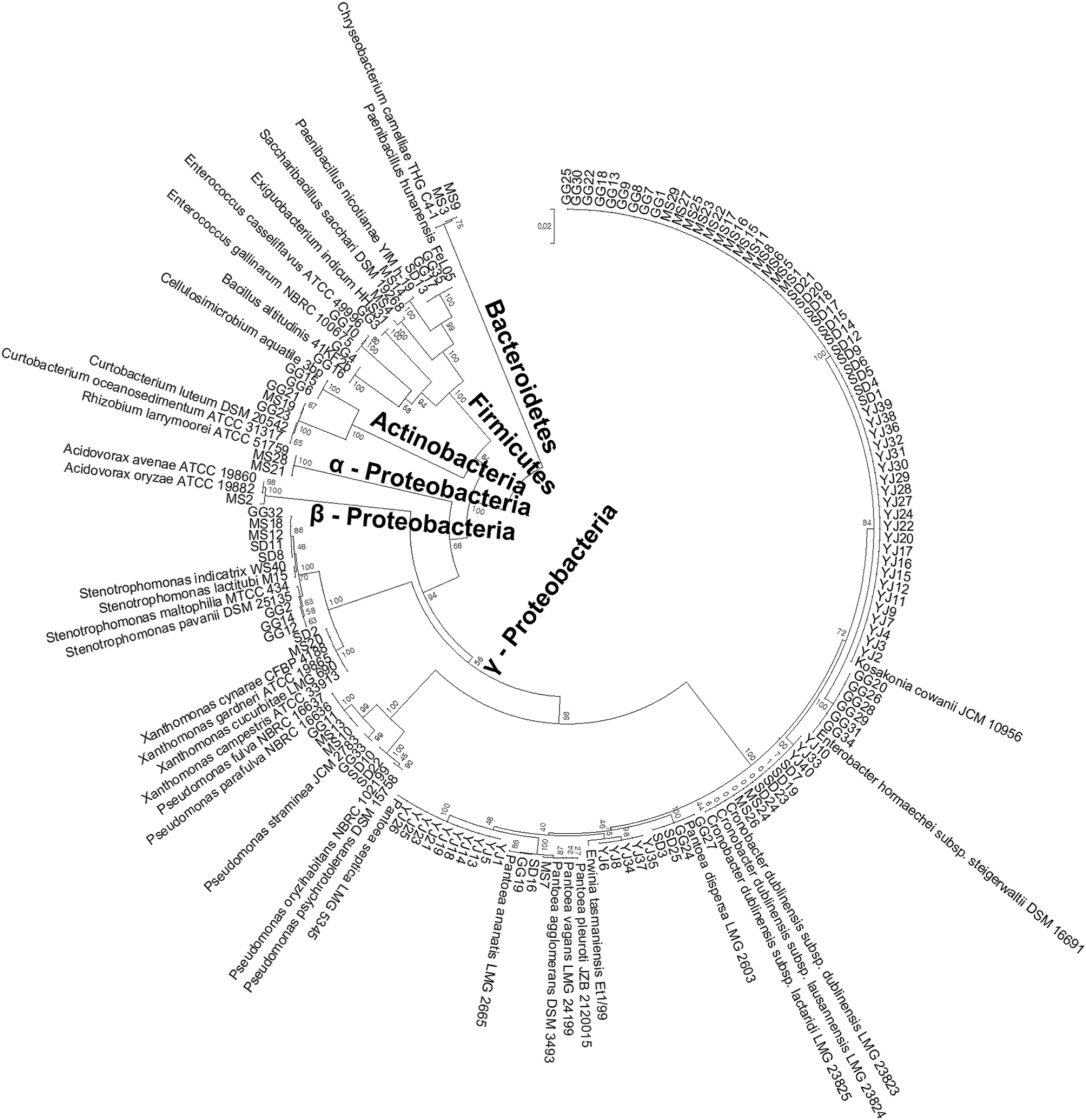
A phylogenetic tree constructed based on the 16S rDNA sequences of seed endophytic bacteria isolated from *Lactuca serriola* seeds and their closely related type strains. A total of 167 nucleotide sequences were analyzed using MEGA software (Version 7.0, https://www.megasoftware.net)^30^. The evolutionary distances were computed using the Kimura 2-parameter method and trees were constructed using the neighbor-joining method. The optimal tree with the sum of branch length = 1.11 is shown. The positions containing gaps and missing data were eliminated. A total of 1,176 positions were included in the final dataset.

**Figure 3:**
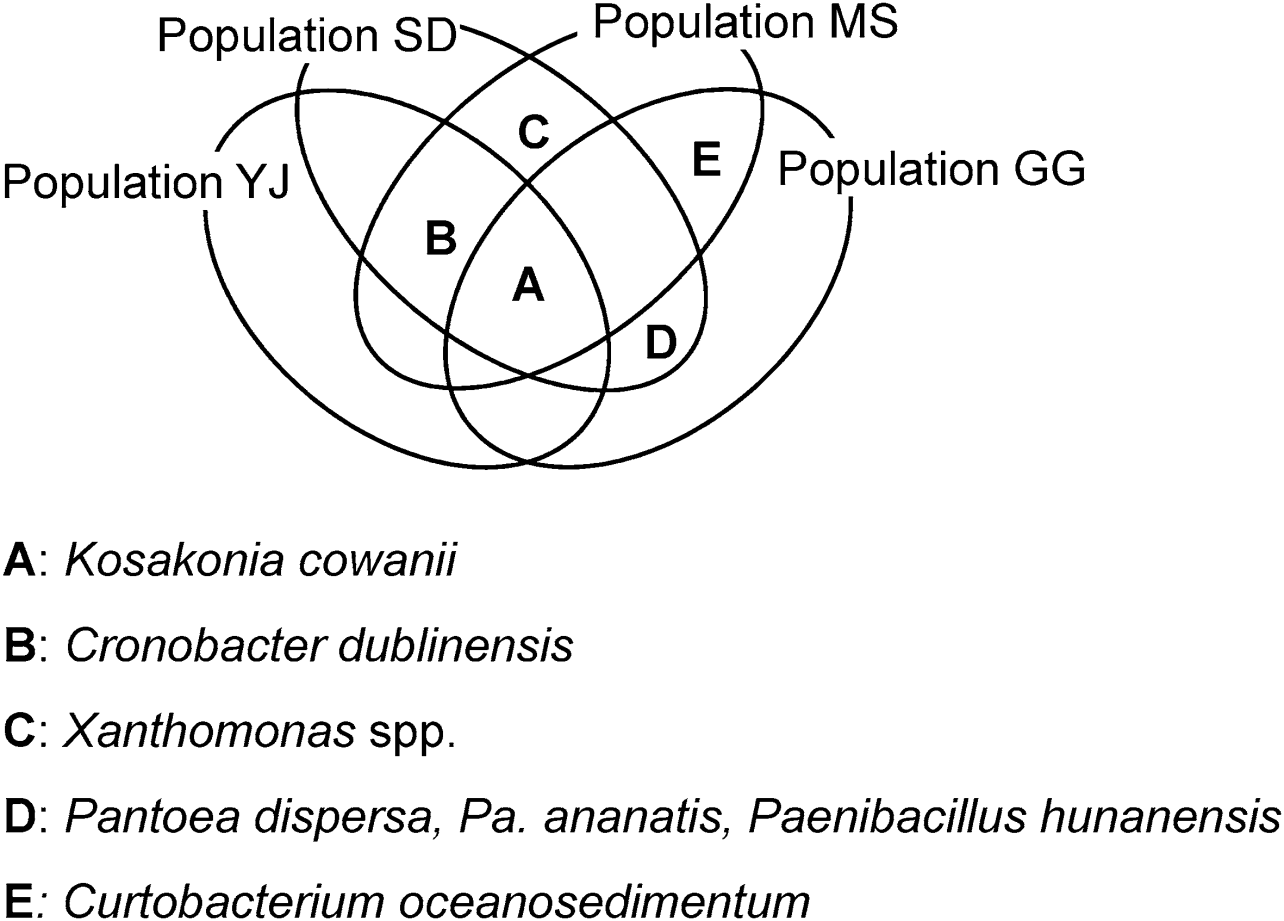
Venn diagram of the shared isolates among the plant populations.

A total of 129 bacterial strains were isolated, and their 16S rDNA sequences were compared with previously reported sequences of bacterial type strains in the EZBioCloud database (Chunlab, Seoul, South Korea). The 16S rDNA sequences of all isolates exhibited more than 99% similarity with the type strains. A total of 18 genera were identified (Fig. 2, Table 1): *Acidovorax*, *Bacillus*, *Cellulosimicrobium*, *Chryseobacterium*, *Cronobacter*, *Curtobacterium*, *Enterobacter*, *Enterococcus*, *Erwinia*, *Exiguobacterium*, *Kosakonia*, *Paenibacillus*, *Pantoea*, *Pseudomonas*, *Rhizobium*, *Saccharibacillus*, *Stenotrophomonas*, and *Xanthomonas*. *K. cowanii* was detected in all four populations, and *Cr. dublinensis* was detected in three populations (Fig. 3). Across all plant populations, most isolates belonged to *Gammaproteobacteria* (Fig. 2). *Firmicutes* constituted 13–25% of the isolates, whereas no *Firmicutes* specimens could be detected in the YJ population. *Alpha*- and *Betaproteobacteria*, *Actinobacteria*, and *Bacteroidetes* were detected in either MS or GG populations, but not in YJ and SD populations.

**Table 1:** Results of the PGP traits in 42 representative isolates.

Isolate	Top-hit strains	PPS	SPP	NF	IAA	ACC	Accession #
YJ1	*Pantoea septica*	+	+	+	+	+	KCTC 72292
YJ4	*Kosakonia cowanii*	+	+	+	+	+	KCTC 72293
YJ6	*Erwinia tasmaniensis*	+	+	+	+	+	KCTC 72294
YJ10	*Cronobacter dublinensis* subsp. *lausannensis*	+	-	+	+	+	KCTC 72295
YJ33	*Cronobacter dublinensis* subspp.	+	+	+	+	+	KCTC 72296
SD1	*Kosakonia cowanii*	+	+	+	+	+	KCTC 72297
SD2	*Xanthomonas* spp.	+	+	+	-	+	KCTC 72298
SD7	*Cronobacter dublinensis* subsp. *lausannensis*	+	+	+	+	+	KCTC 72299
SD8	*Stenotrophomonas maltophilia*	-	+	+	-	+	KCTC 72300
SD10	*Psedomonas* spp.	+	+	+	-	+	KCTC 72301
SD13	*Paenibacillus hunanensis*	+	-	+	+	+	KCTC 43055
SD16	*Pantoea ananatis*	+	+	+	+	+	KCTC 72302
SD25	*Pantoea dispersa*	+	+	+	+	+	KCTC 72303
MS1	*Kosakonia cowanii*	+	+	+	+	+	KCTC 72304
MS2	*Acidovorax* spp.	+	-	+	-	+	KCTC 72291
MS3	*Chryseobacterium camelliae*	-	+	+	+	+	KCTC 72320
MS4	*Saccharibacillus sacchari*	-	-	-	-	+	KCTC 43058
MS7	*Pantoea* spp.	+	+	+	+	+	KCTC 72305
MS12	*Stenotrophomonas* spp.	+	-	+	-	+	KCTC 72306
MS13	*Pseudomonas* spp.	+	-	+	-	+	KCTC 72307
MS14	*Paenibacillus nicotianae*	+	+	+	-	+	KCTC 43057
MS18	*Stenotrophomonas indicatrix*	-	-	+	-	+	KCTC 72308
MS19	*Curtobacterium oceanosedimentum*	+	-	+	-	+	KCTC 49283
MS20	*Xanthomonas* spp.	+	+	+	-	+	KCTC 72309
MS24	*Cronobacter dublinensis* subsp. *dublinensis*	+	+	+	+	+	KCTC 72310
MS26	*Cronobacter dublinensis* subsp. *lausannensis*	+	+	+	+	+	KCTC 72311
MS28	*Rhizobium larrymoorei*	+	+	+	+	+	KCTC 72290
GG1	*Kosakonia cowanii*	+	+	+	-	+	KCTC 72312
GG2	*Stenotrophomonas* spp.	-	-	+	-	+	KCTC 72313
GG3	*Exiguobacterium indicum*	+	-	+	-	+	KCTC 43053
GG4	*Bacillus altitudinis*	+	+	+	-	+	KCTC 43054
GG10	*Enterococcus* spp.	+	+	+	-	+	KCTC 21150
GG11	*Pseudomonas fulva*	+	-	+	-	+	KCTC 72314
GG15	*Cellulosimicrobium aquatile*	+	+	+	-	+	KCTC 49284
GG17	*Paenibacillus hunanensis*	+	-	+	+	+	KCTC 43520
GG19	*Pantoea ananatis*	+	+	+	+	+	KCTC 72319
GG20	*Enterobacter hormaechei* subsp. *steigerwaltii*	+	+	-	+	+	KCTC 72316
GG21	*Curtobacterium oceanosedimentum*	+	+	+	-	+	KCTC 49285
GG23	*Curtobacterium luteum*	+	+	+	-	+	KCTC 49286
GG24	*Pantoea dispersa*	+	+	+	-	+	KCTC 72317
GG32	*Stenotrophomonas lactitubi*	-	+	+	-	+	KCTC 72318
GG33	*Pseudomonas straminea*	+	-	+	-	+	KCTC 72315

Positive and negative results for each trait are indicated by ‘ + ’ and ‘ − ,’ respectively. PPS, Phosphate solubilization; SPP, Siderophore production; NF, Nitrogen fixation; IAA, Indole acetic acid production; ACC, 1-aminocyclopropane-1-carboxylate deaminase production.

PGP traits were examined for 42 representative isolates (Table 1; Supplementary Fig. S1). All tested isolates produced ACC deaminase, and 95.2% of the isolates could grow without a nitrogen source. In addition, 85.7% and 69% of the isolates could solubilize insoluble phosphate and produce siderophores, respectively. Less than half of the isolates produced IAA. IAA production was not observed for *K. cowanii* isolated from the GG site, whereas it was detected for those from other sites. Thus, even though isolates were assigned to the same species based on their 16S rDNA sequence similarity, their PGP activities likely depended on their source population. These 42 isolates have been deposited in the Korean Collection for Type Cultures (KCTC) (Table 1).

### Bacterial growth under drought conditions and EPS production

All 42 representative isolates produced capsular materials outside their cells (Supplementary Fig. S1). When the isolates were grown in media with a water potential of -0.73 MPa, *Pa. ananatis* SD16 presented the highest OD value (1.65), whereas *Pa. nicotianae* MS14 presented the lowest OD value (0.27) (Supplementary Fig. S1). For EPS quantification, *K. cowanii* and the two isolates with the highest OD values were selected from each source population.

**Figure 4:**
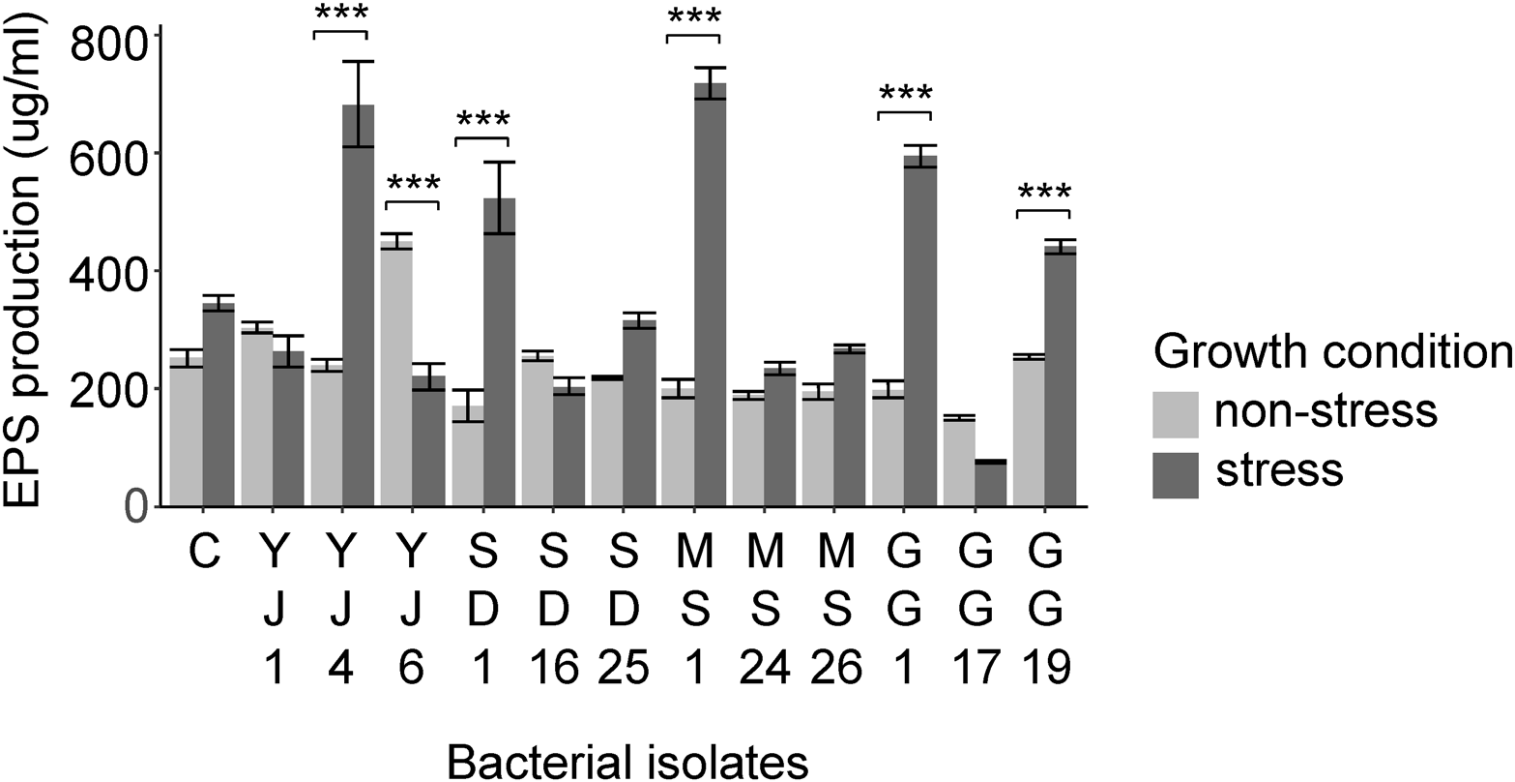
Exopolysaccharides (EPS) production (mean ± SE) of isolates under non-stress and stress conditions, measured in µg/mL. C, *Escherichia coli* DH5a.

The bacterial isolates grown in media with a water potential of − 0.73 MPa tended to produce more EPS than those grown under non-stress condition (F = 8.09, *P* < 0.01). Notably, isolates responded differently to the stress condition, as indicated by the significant isolate × condition interaction (F = 47.62, *P* < 0.001) (Fig. 4). EPS production increased under stress condition in *K. cowanii* YJ4 (t = 6.04, adjusted *P* < 0.001), *K. cowanii* SD1 (t = 5.33, adjusted *P* < 0.001), *K. cowanii* MS1 (t = 17.12, adjusted *P* < 0.001), *K. cowanii* GG1 (t = 16.95, adjusted *P* < 0.001), and *Pa. ananatis* GG19 (t = 13.91, adjusted *P* < 0.001). In contrast, *Erwinia tasmaniensis* YJ6 exhibited lower EPS production under stress condition (t = 9.00, adjusted *P* < 0.001). *K. cowanii* MS1 exhibited the highest EPS production (719.00 µg/mL), whereas *Pa. hunanensis* GG17 presented the lowest EPS production (76.67 µg/mL) under stress condition.

### Effects of isolates on the drought tolerance of *A. thaliana*

**Figure 5:**
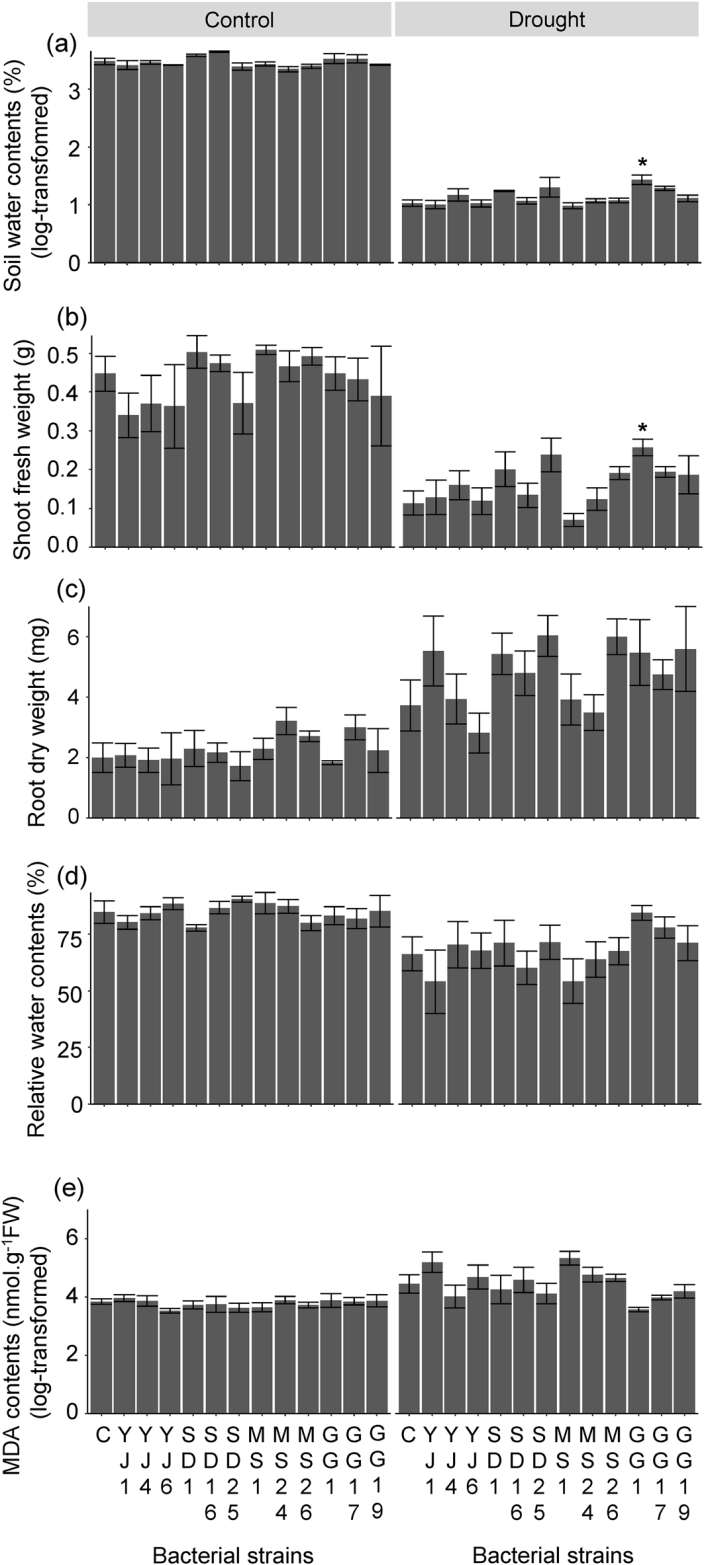
Effects of seed endophytic bacteria on *A. thaliana* growth and soil water content under drought conditions. (**a**) Soil water contents. (**b**) Shoot fresh weight. (**c**) Root dry weight. (**d**) Relative water content. (**e**) Malondialdehyde (MDA) content. Data are presented as mean ± SE. Results of multiple comparisons with Dunnett adjustment are indicated above each bar. *P < 0.05. C: negative control.

After the discontinuation of water supply for 10 days, the soil water content in the drought treatment decreased to 3–5%. Notably, the effects of bacterial treatments on the soil water content differed between the water treatments, as indicated by the significant bacteria × water treatment interaction (F = 2.03, *P* < 0.05). Under moist conditions, the soil water content of the bacteria-inoculated treatments was similar to that of the control without inoculation. In contrast, the water content of soil inoculated with *K. cowanii* GG1 was higher than that of the control without bacteria under drought conditions (t = 3.50, adjusted *P* < 0.05) (Fig. 5a).

**Figure 6:**
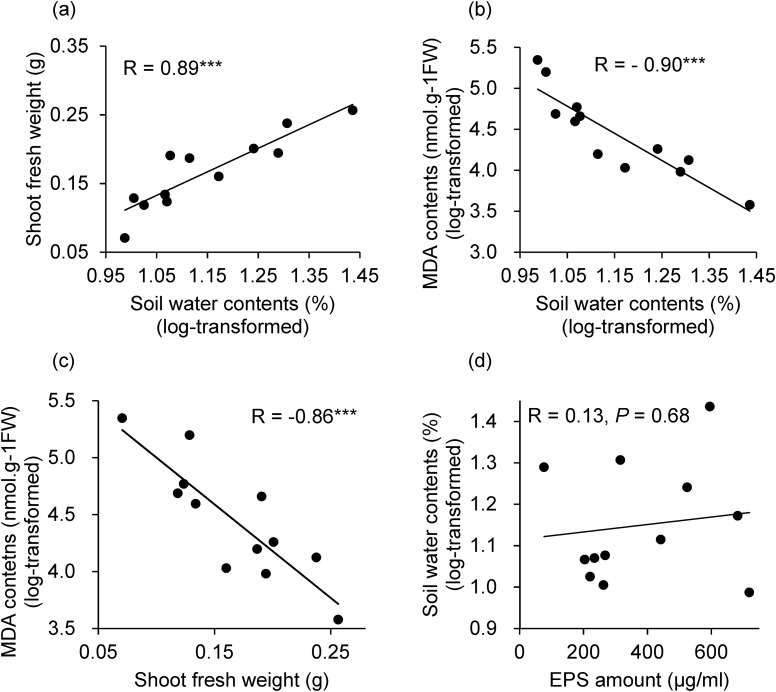
Pearson’s correlation coefficients between the soil water content, shoot fresh weight, malondialdehyde (MDA) concentration, and exopolysaccharides production of isolates.

In plants grown under drought conditions, the bacterial treatment affected shoot fresh weight (F = 2.52, *P* < 0.05) and MDA content (F = 2.62, *P* < 0.05) (Fig. 5b, and e). In particular, *A. thaliana* inoculated with *K. cowanii* GG1 produced heavier shoots (t = 2.97, adjusted *P* < 0.05) than the untreated control (Fig. 5b). Root dry weight (F = 1.56, *P* = 0.14) and relative water content (F = 1.10, *P* = 0.38) did not differ between the bacterial treatments under drought condition (Fig. 5c, and d), but MDA contents did (F = 2.62, *P* = 0.01) (Fig. 5e). The soil water content under drought conditions was positively correlated with the shoot fresh weight and negatively with the MDA concentration (Fig. 6a, b, and c). EPS production in media with water potential of − 0.73 MPa was not correlated with soil water content (Fig. 6d), plant shoot fresh weight, or MDA concentration.

## Discussion

Invasive *L. serriola* plants possess diverse seed endophytic bacteria that produce PGP molecules. Some of them survived and increased EPS production in the growth medium under water stress conditions. When grown under drought conditions, *A. thaliana* plants inoculated with *K. cowanii* GG1 presented a higher shoot biomass than those without bacterial inoculation.

A total of 129 bacterial strains were isolated from *L. serriola* seeds and were assigned to 42 species. All isolated species, except *Ce. aquatile* and *Cr. dublinensis*, have previously been reported as plant-associated bacteria in the rhizosphere, roots, leaves, stems, flowers, or seeds of crop plant species (see Supplementary Table S2 for references). Notably, some of these species have been shown to produce PGP molecules and promote plant growth or stress tolerance. For instance, *Pa. ananatis* increases the root weight of pepper seedlings^31^, and *Pa. dispersa* ameliorates salt tolerance in wheat^32,33^. *Ba. altitudinis* and *En. hormaechei* subsp. *steigerwaltii* increase root length in wheat and maize^34–36^. All 42 representative strains in this study exhibited at least one of the tested PGP traits (Table 1), indicating that the invasive plant *L. serriola* harbors diverse endophytic bacteria with the potential to promote plant growth.

All bacterial isolates produced ACC deaminase. Under environmental stress, the ethylene level increases in plants, resulting in the inhibition of plant growth^37^. ACC is the immediate precursor of ethylene. ACC deaminase cleaves ACC into α-ketobutyrate and ammonia. Thus, bacteria producing ACC deaminase can block ethylene production, which facilitates plant growth under diverse environmental stresses, including drought, salt stress, and flooding^4,38^. The ACC deaminase activity of seed endophytic bacteria might contribute to the stress tolerance of *L. serriola*.

*Proteobacteria*, particularly *Gammaproteobacteria*, was the dominant phylum of isolated endophytes, consistent with the root and leaf microbial communities of a congeneric plant species, *Lactuca sativa*^39,40^. One species of the *Gammaproteobacteria*, *K. cowanii*, was detected in all *L. serriola* populations. *K. cowanii* (*Enterobacter cowanii*) was first isolated from the leaf tissues of *Eucalyptus* plants exhibiting signs of blight disease. Although it is known to cause bacterial spots in *Mabea fistulifera*^41^, endophytic *K. cowanii* isolated from *Tylosema esculentum* exhibited diverse PGP traits^42^. In addition, EPS production by *K. cowanii* was examined for its application as a plant growth promoter^43,44^.

*K. cowanii* isolated in the present study produced more EPS when grown in media with a negative water potential than in control conditions. This is a characteristic of bacterial strains that facilitate plant tolerance to environmental stress^28,29^. *K. cowanii* are known to produce α-type heteropolysaccharides, and the polysaccharide composition depends on growth conditions^43,44^. As observed for *Pseudomonas aeruginosa*, environmental stresses, including drought, can induce the accumulation of diguanylate cyclase, which increases EPS production, although these mechanisms in *K. cowanii* are unknown^45^.

We hypothesized that *L. serriola* seeds might contain bacterial strains that potentially contribute to the drought tolerance of the species. Although bacterial strains isolated from the rhizosphere, roots, and leaves have been shown to promote plant performance under drought stress^46–49^, it is not known if seed endophytic bacteria also have similar activities. In this study, a bacterium isolated from the seeds, *K. cowanii* GG1, improved the aboveground biomass in *A. thaliana* under drought stress conditions, when compared with untreated *A. thaliana*.

The mutualistic relationship between alien plants and microorganisms has been suggested to facilitate or hinder invasive success^13–15^. The development of a novel beneficial association between the pre-existing microorganisms in the introduced habitats and an alien plant promotes the establishment of the alien species. In contrast, invasive success would diminish if no mutualistic microorganisms are available in the introduced habitats. In this context, differential soil microbiota between the native and introduced communities would act as a barrier to the invading alien plant species.

Soil microbiota in the introduced habitats have been postulated as a major source of mutualistic microorganisms, but our results suggest that seed endophytic bacteria might be another source. Microorganisms within seeds of alien plants can disperse with the plant germplasm to novel habitats, and at least some of them, like *K. cowanii* in this study, might be able to promote the plant performance^18–20^. Seed endophytic bacteria, similar to the soil microbiota, might influence the invasive dynamics of alien plants. For instance, if seeds and their beneficial endophytes dispersed simultaneously from their native to the introduced range, seed endophytic bacteria might facilitate the initial establishment of alien plants, a critical step of plant invasion^12^.

Inoculation with *K. cowanii* GG1 increased soil water content under drought conditions as well as the aboveground biomass of *A. thaliana*. Moreover, the soil water content in the bacteria-inoculated treatments was positively correlated with the aboveground biomass and negatively with the MDA content of plants under drought conditions (Fig. 6). These results indicate that isolated bacteria influence soil water retention under drought stress, consequently affecting the production of shoot biomass and controlling physiological damage to the host plants, as indicated by the MDA concentration^46,50^.

As bacterial EPS production likely improves soil water retention^26^, we expected that bacterial EPS production in the media would be positively correlated with the water content of soil with bacterial inoculation. *K. cowanii* GG1 produced a relatively high concentration of EPS when grown in media with a negative water potential, and increased the soil water content under drought conditions when it was used to inoculate the seeds and soil. However, no correlation was detected between the EPS production in the media and soil water content of the tested bacterial strains (Fig. 6). In particular, EPS production by *K. cowanii* MS1 was higher than that of *K. cowanii* GG1; however, the water content of soil inoculated with *K. cowanii* MS1 was lower than that of the control. The EPS production of *P. hunanensis* GG17 was lower than that of *K. cowanii* GG1, whereas the water content of the soil inoculated with these two strains was similar.

Considering the diverse factors that affect bacterial EPS production, the absence of a correlation between bacterial EPS production in the liquid medium and the water content of bacteria-inoculated soil under drought conditions is not surprising. For instance, the amount and composition of EPS are highly dependent on the growth conditions^43,44^. The different conditions of the liquid media used for EPS quantification and the soil used for plant growth likely caused this discrepancy. In addition, bacterial growth in the rhizosphere is known to depend on the host species, likely influenced by the differential chemical compositions of root exudates^51,52^. As we applied the bacteria isolated from *L. serriola* to *A. thaliana*, the effects of these plants on the bacterial growth in the rhizosphere might be different. Lastly, *K. cowanii* strains from plant populations might be distinctive genotypes that might affect plant performance differentially.

A caveat of this study is that we used *A. thaliana* instead of host plant *L. serriola* although model plant species have often been used in similar studies^49,50,53^ and *K. cowanii* GG1 was able to colonize the root of *A. thaliana* (Supplementary Fig. S3). Assessing physiological changes of *L. serriola* in response to the endophyte treatment would be the next step for more complete understanding of invasive host plant–seed endophyte interactions. Transcriptome analysis of *L. serriola* plants and endophytic bacteria will be a useful approach for the physiological study. Metagenome study would provide information on the genetic mechanisms of the effects of endophytic bacteria on plant performances.

In conclusion, *L. serriola*, a xerophytic invasive plant species, possesses diverse seed endophytic bacteria. Notably, one isolate, *K. cowanii* GG1, increased EPS production in media with a highly negative water potential, increased soil water content, and promoted the growth of *A. thaliana* under drought conditions. These results imply that invasive plants can disperse along with their symbiotic bacteria, which may promote successful establishment in the introduced area.

## Methods

### Seed sources and endophyte isolation

We collected wild *L. serriola* seeds from four natural populations in the vicinity of the Gwangju Institute of Sciences and Technology, Gwangju, South Korea (Fig. 1, supplementary table S1). Permission to collect *L. serriola* was given by the Ministry of Environment, Korea. Experimental research on plants including the collection of plant material complied with relevant institutional and national guidelines and legislation. Seeds from 4–12 plants from each population were composited, and 80 seeds from each composite sample were used to isolate endophytic bacteria. To sterilize the seed surface, we removed the seed pappi and soaked the seeds in 70% ethanol for 1 min and 3% sodium hydrochloride for 3 min. The seeds were washed three times with sterile distilled water. To confirm surface sterilization, 100 μL of the rinsing water was spread on R2A (#218,263, Difco) and Luria–Bertani (LB) agar (#7279, Acumedia) and incubated at 25 °C for 7 days.

The surface-sterilized seeds were pulverized using an autoclaved mortar and pestle, and 1 mL of sterile distilled water was added to form a mixture in the clean bench (Hanbaek Co. Ltd, Bucheon, Korea). The mixture (100 μL) was spread on five different solid media, including R2A (#218,263, Difco), potato dextrose agar (PDA; #213,400, Difco), King’s B agar (KB; #60,786, Sigma-Aldrich), LB agar (#7279, Acumedia), and commercial agar. After incubating the plates at 25 ℃ for 1 month, 129 morphologically different colonies were selected. The colonies were subcultured twice and preserved in 20% glycerol stock solutions at -80 ℃ until further analysis.

To extract bacterial genomic DNA, a single colony was inoculated into the LB broth and incubated in a shaking incubator at 25 °C and 180 rpm. DNA was extracted using the Exgene Cell SV kit (GeneAll, Seoul, South Korea) following the manufacturer’s instructions. The 16S rDNA gene was amplified by polymerase chain reaction (PCR) using the universal primers 27F (GAGTTTGATCMTGGCTCAG) and 1492R (TACGGYTACCTTGTTACGACTT), as previously described^54^. Amplification was for 35 cycles in T100 Thermal Cycler (Bio-Rad Laboratories, CA, USA) using the following program: initial denaturation, 95 ℃ for 3 min; denaturation, 95 ℃ for 30 s; annealing, 55 ℃ for 30 s; elongation, 72 ℃ for 1 min; and additional extension for 5 min at 72 ℃. The PCR products were purified using the QIAquick PCR Purification Kit (Qiagen, Venlo, the Netherlands). Sequencing was performed using the Sanger method with universal 16S rDNA 27F and 1492R primer (Macrogen Inc; Seoul, Korea). If required, additional 16S rDNA primers 518F (CCAGCAGCCGCGGTAATACG) and 800R (TACCAGGGTATCTAATCC) were used for sequencing. The nucleotide sequences were aligned using MEGA 7.0 software^30^ and compared with previously reported sequences of bacterial type strains using EZBioCloud (Chunlab, Seoul, South Korea). A phylogenetic tree was constructed using the sequences isolated in the present study and those of the type strains with the highest sequence similarity, using MEGA 7.0. The distance matrix was calculated using Kimura’s two-parameter method, and the phylogenetic tree was constructed using the neighbor-joining method^55,56^. The 16S rDNA sequences of the isolates have been deposited in the NCBI GenBank, and their accession numbers are provided in Table 1.

### Bacterial phenotyping

Of the 129 identified bacterial isolates, 42 representative isolates were selected and their in vitro PGP traits were examined (Supplementary Fig. S1). A representative isolate was randomly selected from a pool of isolated strains in the same phylogenetic clade (Fig. 2). When isolated strains from different plant populations grouped into the same clade, one representative isolate for each population was selected. To examine their ability to solubilize inorganic phosphate, all isolates were inoculated on the National Botanical Research Institute phosphate growth medium (NBRIP) medium [10 g/L glucose, 5 g/L Ca_3_(PO_4_)_2_, 5 g/L MgCl_2_·6H_2_O, 0.25 g/L MgSO_4_·7H_2_O, 0.2 g/L KCl, and 0.1 g/L (NH_4_)_2_SO_4_; 15 g/L agar; pH 7.0] and incubated at 25 °C for 10 days^57^. A clear halo around the colony indicated that the isolate could solubilize inorganic phosphate. To test siderophore production, we prepared 90-mm test plates containing LB and Chrome Azurol S (CAS) media [60.5 mg/L CAS, 72.9 mg/L hexadecyltrimethylammonium bromide (HDTMA), 30.24 g/L Pipes, 10 mL/L iron (III) solution (1 mM FeCl_3_·6H_2_O, 10 mM HCl), and 1:3 (v/v) 0.9% agarose solution] in 1:1 ratio^58^. Each isolate was inoculated at the edge of the LB medium in each test plate and incubated at 25 °C for 1 week. A color change of the CAS medium from blue to orange indicated siderophore production. Indole acetic acid (IAA) production was determined following Johnston-Monje and Raizada^53^. Each isolate was inoculated on LB medium with l g/L of L-tryptophan and incubated at 25 °C for 3 days. A nitrocellulose membrane (Merck Millipore, Darmstadt, Germany) was placed over the agar surface and incubated at 4 °C overnight. Salkowski reagent (0.01 M ferric chloride in 35% perchloric acid; Sigma-Aldrich) was added to the nitrocellulose membrane for 30 min. A pink color indicated IAA production. Nitrogen fixation and 1-aminocyclopropane-1-carboxylate (ACC) deaminase production were tested by culturing each isolate on DF salt agar medium without a nitrogen source (4 g/L KH_2_PO_4_, 6 g/L Na_2_HPO_4_, 0.2 g/L MgSO_4_․7H_2_O, 1.0 mg/L FeSO_4_․7H_2_O, 1.0 mg/L H_3_BO_3_, 10 µg/L MnSO_4_, 70 µg/L ZnSO_4_, 50 µg/L CuSO_4_, 10 µg/L MoO_3_, and 15 g/L agar), without or with 2 mM ACC, respectively. Bacterial growth after incubation at 25 °C for 5 days indicated nitrogen fixation or ACC deaminase activity^59^.

### Screening for drought-tolerant bacteria

Before examining the drought tolerance of the isolated bacteria, we tested if the isolates produced capsular material outside the cells as a quick screening procedure of drought-tolerant bacteria^60^. All isolates cultured on the KB medium were stained using a negative capsule staining method with 10% nigrosine and 1% crystal violet^61^. We observed clear halo zones around all isolated bacterial cells, indicating capsular material production (Supplementary Fig. S1).

To examine the drought tolerance of the 42 selected isolates, we measured their growth in trypticase soya broth (TSB; #211,825, Difco) with a water potential of -0.73 MPa following Sandhya et al.^28,62^. The water potentials were adjusted with 25% polyethylene glycol 6000 (#817,007, Merck), as described by Michel and Kaufmann^63^. After incubating the bacterial isolates in TSB overnight, 1% volume aliquots of the incubated cultures were transferred to TSB with -0.73 MPa and incubated in a shaking incubator at 28 °C and 180 rpm for 24 h. The growth of each bacterial isolate was estimated by measuring the optical density of the sample at 600 nm (OD_600_) using BioSpectrometer basic (Eppendorf, Hamburg, Germany). The *Escherichia coli* DH5α strain was used as a control to confirm the experimental protocol of the drought tolerance test and the EPS quantification because this strain is known to produce colonic acid, a kind of EPS^64^. The OD_600_ of triplicate samples was measured for each isolate.

### Quantification of EPS in drought-tolerant bacteria

Of the seed endophytes isolated from each plant population, we selected the two isolates with the highest OD_600_ values at − 0.73 MPa and one isolate (*Kosakonia cowanii*) detected in all four plant populations for EPS quantification. A total of 12 bacterial isolates were incubated under non-stress (TSB) and stress (TSB at -0.73 MPa) conditions and EPS production was measured following Liu et al.^65^. After culturing the isolates at 28 °C with shaking at 180 rpm for 3 days, the bacterial cells were harvested by centrifugation at 9,000 rpm for 15 min. The bacterial pellet was dissolved in sterilized deionized water for the measurement of dry bacterial biomass. Three volumes of cold ethanol were added to the separated supernatant and the samples were incubated at 4 °C overnight to facilitate EPS precipitation. After centrifugation at 15,000 rpm for 30 min at 4 °C, the precipitated EPS were washed twice with cold absolute ethanol and dissolved in heated deionized water. The concentration of the completely dissolved EPS was measured by the phenol–sulfuric acid method, using D-glucose as a standard^66^.

### Effects of isolates on the growth of *A. thaliana* under the drought condition

To examine the effects of seed endophytic bacteria on plant performance, the model plants, i.e., *A. thaliana* ecotype Col-0, were inoculated twice with 12 isolates, and their EPS production was quantified. After cold treatment at 4 °C for 1 week, surface-sterilized *A. thaliana* seeds were immersed in a bacterial suspension in phosphate-buffered saline (PBS; 10^8^ CFU mL^−1^) for 30 min^67^. Seeds submerged in PBS were used as a negative control. The infected seeds were sown in pots (8 cm × 7.5 cm × 6 cm) containing a 3:1 (volume) blend of sterile vermiculite (Green Fire Chemicals, Hongseong, Korea) and sand (Glpark, Seoul, Korea)^46^. Three weeks after sowing, a bacterial suspension (3.8 × 10^5^ CFU g soil^−1^) in Hoagland’s solution [1.25 mM KNO_3_, 1.5 mM Ca(NO_3_)_2_, 0.75 mM MgSO_4_, 0.5 mM KH_2_PO_4_, 0.05 mM H_3_BO_3_, 0.01 mM MnCl_2_, 0.002 mM ZnSO_4_, 0.0015 mM CuSO_4_, 0.075 μM NH_4_Mo_7_O_24_, and 0.074 mM Fe-EDTA] was applied to each pot near the plant’s roots^46^. The same amount of Hoagland’s solution without bacteria was used as a negative control.

Ten plants were randomly assigned to each bacterial treatment. The pots were randomly positioned in a growth chamber (Hanbaek, Co. LTD., Kyunggi-do, South Korea) and maintained at 22 °C under a 16/8 h light/dark photoperiod throughout the experiment. Sterilized deionized water was applied to each pot once or twice per week, and 20 mL of Hoagland’s solution was applied to each pot once per week during the growth^46^. Half of the plants were subjected to drought conditions 5 weeks after sowing by discontinuing their watering.

After 10 days of drought treatment, the plants were harvested and shoot fresh weight was measured immediately. The roots were dried in a drying oven (Hanbaek Co. LTD., Kyunggi-do, South Korea) at 70 °C for 72 h and their dry weight was measured. The leaves were frozen in liquid nitrogen and stored at -80 °C to measure their malondialdehyde (MDA) content, following Zhang and Huang^68^. In response to drought stress, MDA accumulates in plant leaves as a byproduct of oxidative damage to membrane lipids. The leaves were homogenized in 0.1% (w/v) trichloroacetic acid (TCA; T6399, Sigma-Aldrich), and the mixture was reacted with 20% TCA containing 0.5% thiobarbituric acid (TBA; T5500, Sigma-Aldrich) by boiling at 95 °C for 15 min. The absorbance of the resulting mixture was measured at 532 nm using BioSpectrometer basic (Eppendorf). The relative water content of the plants was determined following the methods of Türkan et al.^69^. Leaf fresh weight was measured immediately after harvesting. Leaf turgid weight was measured after submersion in sterilized deionized water for 24 h at 4 °C in a refrigerator, and the dry weight was measured after drying at 70 °C for 72 h. The gravimetric soil water content at the time of harvest was measured after drying the soil at 105 ℃ for 72 h.

### Statistical analysis

All statistical analyses were performed using R software 4.0.1 (R Foundation for Statistical Computing, Austria). To compare the EPS production of the isolated bacteria, a two-way analysis of variance (ANOVA) was performed, with bacterial isolates, stress conditions, and their interaction as independent variables and EPS production as the dependent variable. Pair-wise differences between the stress conditions of each isolate were evaluated based on the Bonferroni method. To assess the effect of bacterial isolates on plant performance and soil water content, a two-way ANOVA was used, with the drought treatment, isolates, and their interaction as independent variables, and plant traits and soil water content as the dependent variables. Soil water and MDA content was log-transformed to normalize the data, and outliers for all measured traits were excluded from the dataset. As bacterial effects on soil moisture depend on soil water conditions (see results), the control and drought treatments were further assessed separately using ANOVA. Differences between the effects of the negative control and those of the isolated bacteria were evaluated based on Dunnett’s adjustment, under control and drought conditions. To determine the relationship between the EPS production of isolates, soil water content, and plant traits, Pearson’s correlation coefficients were calculated.

## Supplementary Information

Supplementary Information.
