# Supplementary material for: Invasive Lactuca serriola seeds contain endophytic bacteria that contribute to drought tolerance
Source: Sci Rep. 2021 Jun 25;11:13307. doi: 10.1038/s41598-021-92706-x (PMC8233371; doi:10.1038/s41598-021-92706-x)
Supplement: Supplementary file 1 — Supplementary Information. [file 41598_2021_92706_MOESM1_ESM.docx]

**Title:** Invasive *Lactuca serriola* seeds contain endophytic bacteria that contribute to drought tolerance

**Journal name:** Scientific Reports

**Author names:** Seorin Jeong, Tae-Min Kim, Byungwook Choi, Yousuk Kim, and Eunsuk Kim

**Affiliation:** School of Earth Sciences and Environmental Engineering, Gwangju Institute of Science and Technology, Gwangju, South Korea

**Corresponding author:** Eunsuk Kim, Email: eunsukkim@gist.ac.kr

Figure S1. Examples of the plant growth-promoting (PGP) test and capsule staining. The upper pictures illustrate negative results and the lower pictures illustrate positive results in the PGP test. The magnification was 400× and 1000× for capsule staining. (a) Phosphate solubilization. The negative result for *Stenotrophomonas* spp. GG32 and the positive result for *Kosakonia cowanii* SD1. (b) Siderophore production. The negative result for *Paenibacillus hunanensis* SD13 and the positive result for *Cronobacter dublinensis* subsp. *dublinensis* MS24. (c) IAA production. The negative result for *K. cowanii* GG1 and the positive result for *Cr. dublinensis* subsp. *dublinensis* MS24. (d) Capsule staining of *Pseudomonas fulva* GG11. (Upper) 400 X magnification (Lower) 1000 X magnification.

**
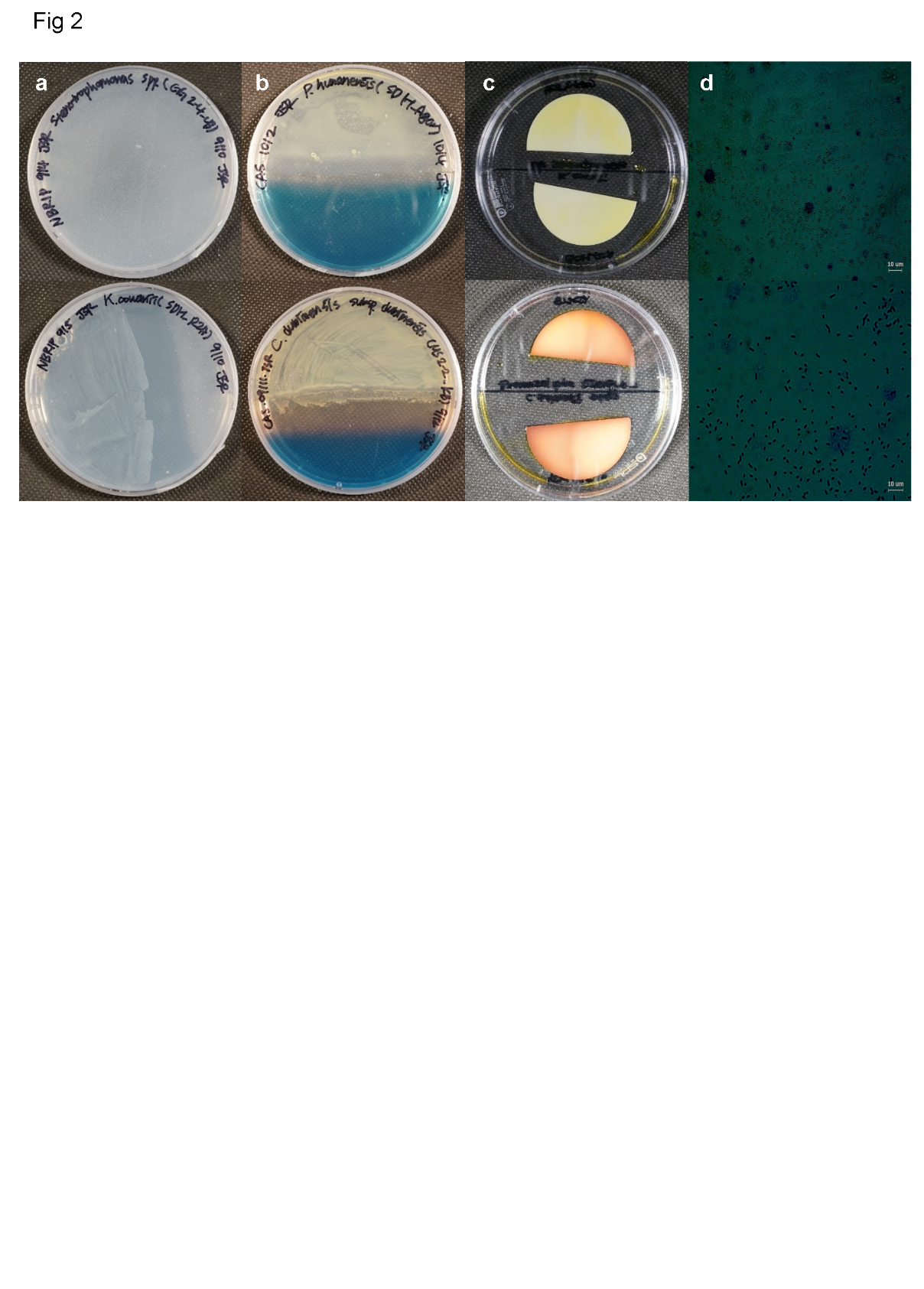
**

Figure S2. OD value (mean ± SE) of isolates in TSB at -0.73 MPa. Species names of the tested isolates are listed in Table 1. Each color represents a source population. C, *Escherichia coli* DH5a.

Figure S3. Image under LSM 880 NLO upright two-photon confocal microscope (ZEISS; NY, USA) of *A. thaliana* root after three weeks of *K. cowanii* GG1 treatment. *K. cowanii* GG1 was tagged with GFPuv by electroporation using pDSK-GFPuv plasmid (#PVT17263, Nova lifetech; Caspian, Singapore)^1^. To diminish chlorophyll autofluorescence, plant tissue was cleared with ClearSee (#031-25251, FUJIFILM; Tokyo, Japan) and then stained with Calcofluor White for the cell wall observation^2^. The cell wall was detected with emission at 405 nm and excitation at 446 nm, while GFP signal was detected with emission at 488 nm and excitation at 516 nm. The image was taken under 50 x magnification, and *K. cowanii* GG1-*gfp* was detected at the tip of the root hair (arrow).

**
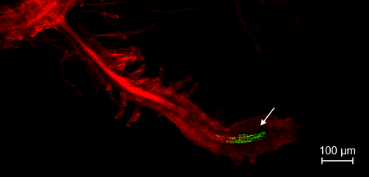
**

1. Wang, K., Kang, L., Anand, A., Lazarovits, G. & Mysore, K. S. Monitoring in planta bacterial infection at both cellular and whole‐plant levels using the green fluorescent protein variant GFPuv. New Phytologist 174, 212-223 (2007).

2. Ursache, R., Andersen, T. G., Marhavý, P. & Geldner, N. A protocol for combining fluorescent proteins with histological stains for diverse cell wall components. The Plant Journal 93, 399-412 (2018).

Table S1. Source populations of *Lactuca serriola* with their locations

| **Population** | **Latitude** | **Longitude** |
| --- | --- | --- |
| Yongjeong (YJ) | 34° 58' 35.688'' N | 127° 29' 1.896'' E |
| Seongdong (SD) | 34° 50' 16.26'' N | 127° 26' 24.108'' E |
| Masan-ri (MS) | 34° 59' 39.372'' N | 126° 28' 59.34'' E |
| Gagok (GG) | 35° 2' 58.668'' N | 126° 22' 51.78'' E |

Table S2. Species names with highest 16S rDNA sequence similarity with isolates in this study. References for those species are given.

| Isolated species | References |
| --- | --- |
| *Acidovorax avenae* | Sturz et al. (1999) |
| *Acidovorax oryzae* | Pereira and Castro (2014) |
| *Bacillus altitudinis* | Compant et al. (2011) |
| *Cellulosimicrobium aquatile* | Eida et al. (2020) |
| *Chryseobacterium camelliae* | Kook et al. (2014) |
| *Cronobacter dublinensis* subsp. *dublinensis* | Mane and Hamde (2018) |
| *Cronobacter dublinensis* subsp. *lausannensis* | - |
| *Curtobacterium luteum* | Sturz et al. (1999) |
| *Curtobacterium oceanosedimentum* | Chen et al. (2012); Pereira and Castro (2014) |
| *Enterobacter hormaechei* subsp. *steigerwaltii* | Egamberdieva et al. (2008) |
| *Enterococcus casseliflavus* | Afzal et al. (2015) |
| *Enterococcus gallinarum* | Mussa et al. (2018) |
| *Erwinia tasmaniensis* | Geider et al. (2006) |
| *Exiguobacterium indicum* | Afzal et al. (2015) |
| *Kosakonia cowanii* | Chimwamurombe et al. (2016) |
| *Paenibacillus hunanensis* | Liu et al. (2010) |
| *Paenibacillus nicotiane* | Li et al. (2014) |
| *Pantoea agglomerans* | Verma et al. (2018) |
| *Pantoea ananatis* | Gagne‐Bourgue et al. (2013) |
| *Pantoea dispersa* | Y. Chen et al. (2014) |
| *Pantoea pleuroti* | - |
| *Pantoea septica* | Fridman et al. (2012) |
| *Pantoea vagans* | Afzal et al. (2015) |
| *Pseudomonas fulva* | Sandhya et al. (2017) |
| *Pseudomonas oryzihabitans* | Verma et al. (2018) |
| *Pseudomonas psychrotolerans* | L. Chen et al. (2014) |
| *Pseudomonas straminea* | Goh et al. (2015) |
| *Rhizobium larrymoorei* | T. Chen et al. (2014) |
| *Saccharibacillus sacchari* | Rivas et al. (2008) |
| *Stenotrophomonas indicatrix* | - |
| *Stenotrophomonas lactitubi* | - |
| *Stenotrophomonas maltophilia* | Alavi et al. (2013) |
| *Stenotrophomonas pavanii* | Feng et al. (2017) |
| *Xanthomonas campestris* pv. *campestris* | Jelušić et al. (2020) |
| *Xanthomonas cucurbitae* | Zhang and Babadoost (2018) |
| *Xanthomonas cynarae* | Han et al. (2009) |
| *Xanthomonas gardneri* | Afzal et al. (2015) |

**References**

Afzal, I., Shinwari, Z. K., & Iqrar, I. (2015). Selective isolation and characterization of agriculturally beneficial endophytic bacteria from wild hemp using canola. *Pak J Bot, 47*(5), 1999-2008.

Alavi, P., Müller, H., Cardinale, M., Zachow, C., Sánchez, M. B., Martínez, J. L., & Berg, G. (2013). The DSF quorum sensing system controls the positive influence of Stenotrophomonas maltophilia on plants. *PLoS One, 8*(7), e67103.

Chen, L., Xu, M., Zheng, Y., Men, Y., Sheng, J., & Shen, L. (2014). Growth promotion and induction of antioxidant system of tomato seedlings (Solanum lycopersicum L.) by endophyte TPs-04 under low night temperature. *Scientia Horticulturae, 176*, 143-150.

Chen, T., Chen, Z., Ma, G., Du, B., Shen, B., Ding, Y., & Xu, K. (2014). Diversity and potential application of endophytic bacteria in ginger. *Genet Mol Res, 13*(3), 4918-4931.

Chen, W.-M., Tang, Y.-Q., Mori, K., & Wu, X.-L. (2012). Distribution of culturable endophytic bacteria in aquatic plants and their potential for bioremediation in polluted waters. *Aquatic Biology, 15*(2), 99-110.

Chen, Y., Fan, J.-B., Du, L., Xu, H., Zhang, Q.-H., & He, Y.-Q. (2014). The application of phosphate solubilizing endophyte Pantoea dispersa triggers the microbial community in red acidic soil. *Applied soil ecology, 84*, 235-244.

Chimwamurombe, P. M., Grönemeyer, J. L., & Reinhold-Hurek, B. (2016). Isolation and characterization of culturable seed-associated bacterial endophytes from gnotobiotically grown Marama bean seedlings. *FEMS Microbiology Ecology, 92*(6), fiw083.

Compant, S., Mitter, B., Colli-Mull, J. G., Gangl, H., & Sessitsch, A. (2011). Endophytes of grapevine flowers, berries, and seeds: identification of cultivable bacteria, comparison with other plant parts, and visualization of niches of colonization. *Microbial ecology, 62*(1), 188-197.

Egamberdieva, D., Kamilova, F., Validov, S., Gafurova, L., Kucharova, Z., & Lugtenberg, B. (2008). High incidence of plant growth‐stimulating bacteria associated with the rhizosphere of wheat grown on salinated soil in Uzbekistan. *Environmental microbiology, 10*(1), 1-9.

Eida, A. A., Bougouffa, S., Alam, I., Saad, M. M., & Hirt, H. (2020). Complete genome sequence of the endophytic bacterium Cellulosimicrobium sp. JZ28 isolated from the root endosphere of the perennial desert tussock grass Panicum turgidum. *Archives of microbiology*, 1-7.

Feng, F., Ge, J., Li, Y., He, S., Zhong, J., Liu, X., & Yu, X. (2017). Enhanced degradation of chlorpyrifos in rice (Oryza sativa L.) by five strains of endophytic bacteria and their plant growth promotional ability. *Chemosphere, 184*, 505-513.

Fridman, S., Izhaki, I., Gerchman, Y., & Halpern, M. (2012). Bacterial communities in floral nectar. *Environmental Microbiology Reports, 4*(1), 97-104.

Gagne‐Bourgue, F., Aliferis, K., Seguin, P., Rani, M., Samson, R., & Jabaji, S. (2013). Isolation and characterization of indigenous endophytic bacteria associated with leaves of switchgrass (P anicum virgatum L.) cultivars. *Journal of applied microbiology, 114*(3), 836-853.

Geider, K., Auling, G., Du, Z., Jakovljevic, V., Jock, S., & Völksch, B. (2006). Erwinia tasmaniensis sp. nov., a non-phytopathogenic bacterium from apple and pear trees. *International journal of systematic and evolutionary microbiology, 56*(12), 2937-2943.

Goh, J. K., Ting, A. S. Y., Lim, P. S., & Adewale, A. M. (2015). Antimicrobial and enzymatic activities of endophytic bacteria isolated from Mentha spicata (MINT). *Malaysian Journal of Microbiology, 11*(2), 102-108.

Han, J., Xia, D., Li, L., Sun, L., Yang, K., & Zhang, L. (2009). Diversity of culturable bacteria isolated from root domains of moso bamboo (Phyllostachys edulis). *Microbial ecology, 58*(2), 363-373.

Jelušić, A., Berić, T., Mitrović, P., Dimkić, I., Stanković, S., Marjanović‐Jeromela, A., & Popović, T. (2020). New insights into the genetic diversity of Xanthomonas campestris pv. campestris isolates from winter oilseed rape in Serbia. *Plant Pathology*.

Kook, M., Son, H.-M., Ngo, H. T., & Yi, T.-H. (2014). Chryseobacterium camelliae sp. nov., isolated from green tea. *International journal of systematic and evolutionary microbiology, 64*(3), 851-857.

Li, Q.-Q., Zhou, X.-K., Dang, L.-Z., Cheng, J., Hozzein, W. N., Liu, M.-J., Hu, Q., Li, W.-J., & Duan, Y.-Q. (2014). Paenibacillus nicotianae sp. nov., isolated from a tobacco sample. *Antonie Van Leeuwenhoek, 106*(6), 1199-1205.

Liu, Y., Liu, L., Qiu, F., Schumann, P., Shi, Y., Zou, Y., Zhang, X., & Song, W. (2010). Paenibacillus hunanensis sp. nov., isolated from rice seeds. *International journal of systematic and evolutionary microbiology, 60*(6), 1266-1270.

Mane, G. G., & Hamde, V. S. (2018). Isolation and identification of Exopolysaccharide producing Cronobacter species from root nodules of leguminous plants. *Journal of Biochemical Technology, 9*(2), 1.

Mussa, A., Million, T., & Assefa, F. (2018). Rhizospheric bacterial isolates of grass pea (Lathyrus sativus L.) endowed with multiple plant growth promoting traits. *Journal of applied microbiology, 125*(6), 1786-1801.

Pereira, S., & Castro, P. (2014). Diversity and characterization of culturable bacterial endophytes from Zea mays and their potential as plant growth-promoting agents in metal-degraded soils. *Environmental Science and Pollution Research, 21*(24), 14110-14123.

Rivas, R., Garcia-Fraile, P., Zurdo-Pineiro, J. L., Mateos, P. F., Martinez-Molina, E., Bedmar, E. J., Sanchez-Raya, J., & Velazquez, E. (2008). Saccharibacillus sacchari gen. nov., sp. nov., isolated from sugar cane. *International journal of systematic and evolutionary microbiology, 58*(8), 1850-1854.

Sandhya, V., Shrivastava, M., Ali, S. Z., & Prasad, V. S. S. K. (2017). Endophytes from maize with plant growth promotion and biocontrol activity under drought stress. *Russian agricultural sciences, 43*(1), 22-34.

Sturz, A., Christie, B., Matheson, B., Arsenault, W., & Buchanan, N. (1999). Endophytic bacterial communities in the periderm of potato tubers and their potential to improve resistance to soil-borne plant pathogens. *Plant Pathology, 48*(3), 360-369.

Verma, S. K., Kingsley, K., Bergen, M., English, C., Elmore, M., Kharwar, R. N., & White, J. F. (2018). Bacterial endophytes from rice cut grass (Leersia oryzoides L.) increase growth, promote root gravitropic response, stimulate root hair formation, and protect rice seedlings from disease. *Plant and Soil, 422*(1-2), 223-238.

Zhang, X., & Babadoost, M. (2018). Characteristics of Xanthomonas cucurbitae Isolates from Pumpkins and Survival of the Bacterium in Pumpkin Seeds. *Plant disease, 102*(9), 1779-1784.
